# Ciprofloxacin‐Induced Near‐Total Toxic Epidermal Necrolysis Complicated by Upper Gastrointestinal Bleeding: A Case Report With ALDEN‐Based Causality Assessment

**DOI:** 10.1002/ccr3.73265

**Published:** 2026-07-30

**Authors:** Amanuel D. Wakoya, Alemu B. Mesekere, Seid A. abdullahi, Misale B. Tesfaye, Zeynu H. Endris, Zelalem G. Demissie, Tibebu G. Haile

**Affiliations:** ^1^ Department of Intensive Care Medicine Saint Paul's Hospital Millennium Medical College Addis Ababa Ethiopia; ^2^ Department of Emergency and Critical Care Medicine Haramaya University Harar Ethiopia

**Keywords:** adverse drug reaction, ALDEN score, ciprofloxacin, gastrointestinal bleeding, toxic epidermal necrolysis

## Abstract

Toxic epidermal necrolysis (TEN) is a rare but life‐threatening mucocutaneous adverse drug reaction characterized by widespread epidermal necrosis and detachment with significant mucosal involvement. We report a case of near total (80%–90%) body surface area TEN in a previously healthy 30‐year‐old man that developed approximately 5 days after completing a 5‐day course of oral ciprofloxacin. The presentation was complicated by upper gastrointestinal bleeding with transient hemodynamic instability. Prompt withdrawal of the offending drug, aggressive supportive care in the intensive care unit, wound management, and multidisciplinary care led to successful recovery. Causality was assessed using the ALDEN (Algorithm of Drug Causality for Epidermal Necrolysis) score, which supported a probable association with ciprofloxacin. This case emphasizes the need for vigilance regarding fluoroquinolone‐associated severe cutaneous adverse reactions even after short‐term use, and the utility of structured causality assessment tools.

## Key Clinical Message

Ciprofloxacin, although rarely implicated, can cause severe toxic epidermal necrolysis. Early recognition, prompt drug withdrawal, and structured causality assessment using the ALDEN algorithm are essential to improve outcomes, particularly in resource‐limited settings.

**Abbreviations**
ALDEN: Algorithm of Drug Causality for Epidermal Necrolysis
BSA: Body surface area
SCARS: severe cutaneous adverse reactions
TEN/SJS: Toxic epidermal necrolysis (TEN) and stevens‐johnson syndrome (SJS)

## 1 Introduction

Toxic epidermal necrolysis (TEN) and stevens‐johnson syndrome (SJS) represent a spectrum of severe cutaneous adverse reactions (SCARS) characterized by wide spread keratinocyte apoptosis, epidermal detachment, and mucosal involvement. TEN is defined by by > 30% body surface area (BSA) detachment and carries a mortality rate of 20%–40%, influenced by age, comorbidities, and extent of involvement [1]. Medications are the predominant trigger with common culprits including allopurinol, sulfonamides, aromatic anticonvulsants, and certain antibiotics. Fluoroquinolones such as ciprofloxacin have been implicated rarely in case reports of SJS/TEN, though they are less frequently cited as high‐risk compared to other classes. Early withdrawal of the agent, supportive care in a specialized unit (often burn/ICU), and multidisciplinary input are cornerstones of management [2]. Systemic corticosteroids remain controversial but are used in some settings: other modalities include IVIG or cyclosporine in selected case. We report a case of near total TEN in 30 year old man following ciprofloxacin exposure, complicated by upper gastrointestinal bleeding and transient hemodynamic instability, who improved with intensive supportive treatment and close monitoring.

## 2 Case History and Examination

**FIGURE 1:**
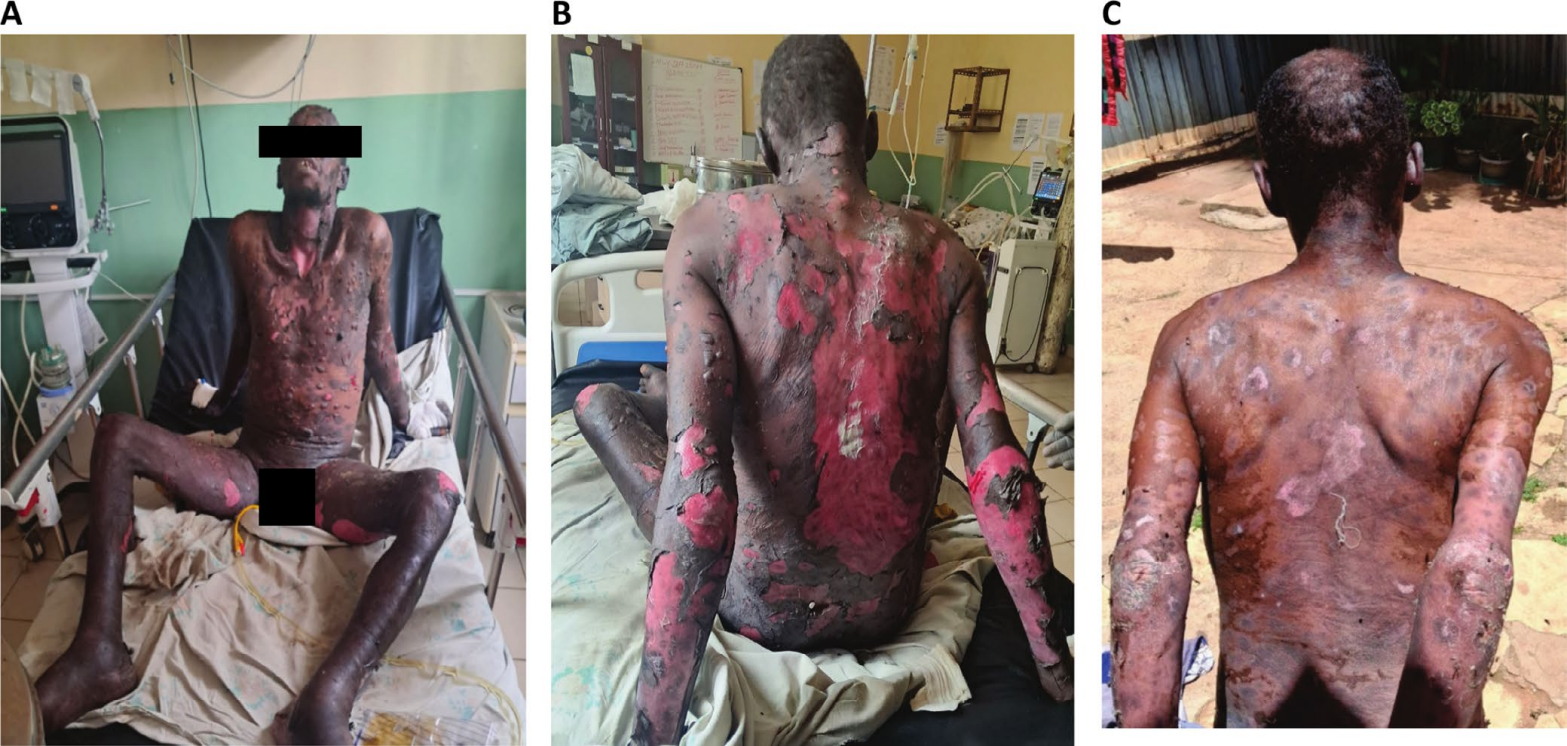
Clinical progression of toxic epidermal necrolysis. (A) Extensive epidermal detachment involving the anterior and posterior body surfaces at presentation. (B) Widespread denuded skin and persistent lesions during intensive care unit admission (Day 7). (C) Clinical improvement by Day 16, showing resolving denuded areas with patchy post‐inflammatory hyperpigmentation and no active blistering.

A 30‐year‐old Ethiopian man was referred to our hospital with extensive erosive skin lesions involving nearly the entire body. Detailed history revealed a progressive mucocutaneous rash following completion of a 5‐day course of oral ciprofloxacin 500 mg twice daily, which had been prescribed at a local health center for back pain, abdominal pain, and headache. Five days after discontinuing the medication, he developed oral mucosal lesions and a generalized macular rash that progressed to diffuse skin darkening and widespread epidermal detachment over three days (Figure 1). He also reported fever, malaise, and frequent episodes of bloody vomiting prior to presentation, with three additional episodes after arrival. A chronological summary of the patient's clinical course is presented in Table 1.

**TABLE 1:** Chronology of symptom onset, clinical deterioration, treatment, and early in‐hospital outcome.

Time point	Clinical details
Before presentation	Back pain, abdominal pain, and headache treated with oral ciprofloxacin 500 mg twice daily for 5 days.
5 days after stopping ciprofloxacin	Oral mucosal lesions and generalized macular rash appeared.
Progression before hospital arrival	Rash progressed to diffuse skin darkening and widespread epidermal detachment; fever, malaise, and many episodes of bloody vomiting developed and three more in hospital.
Day of admission	In pain, BP 90/56 mmHg, pulse 134 beats/min, respiratory rate 36 breaths/min, temperature 34°C, SpO2 94% on room air; 80%–90% body surface area involvement, positive Nikolsky sign, ocular and mucosal involvement.
Initial management	Intravenous fluid resuscitation, omeprazole loading dose and maintenance therapy, ICU transfer, hydrocortisone, wound care, opioid analgesia, nasogastric feeding, and eye care.
Hospital day 3	Recurrent hypotension (88/50 mmHg), persistent tachycardia, and fever (38.4°C); empirical cefepime and vancomycin started for suspected sepsis pending repeat investigations.
Clinical course/outcome	Stayed in ICU for 5 days, then improved clinically with progressive resolution of blistering, residual patchy hyperpigmentation, stable vital signs, adequate urine output, and improved laboratory findings and transferred to ward.

The patient used paracetamol for symptomatic relief of headache and fever and reported prior tolerance without adverse reactions. He denied use of any other co‐ingested medications, herbal remedies, or supplements. He had no known chronic medical illness and no personal or family history of similar reactions or significant allergy.

On arrival at the emergency department, he appeared acutely ill and in pain. His blood pressure was 90/56 mmHg, pulse rate 134 beats/min, respiratory rate 36 breaths/min, temperature 34°C, and oxygen saturation 94% on room air. Skin examination demonstrated extensive epidermal detachment involving approximately 80%–90% of the body surface area, with dusky necrotic patches, blisters, and large denuded areas. Nikolsky sign was positive, and both ocular and mucosal involvement were present.

## 3 Methods (Differential Diagnosis, Investigations and Treatment)

Based on the temporal relationship to drug exposure and the clinical findings, a working diagnosis of ciprofloxacin‐induced toxic epidermal necrolysis was made. Formal causality assessment using the ALDEN algorithm later confirmed a probable association (Table 2).

**TABLE 2:** ALDEN score for ciprofloxacin.

Criterion	Details	Score
Delay from initial intake to onset	~5 days use + ~7 days after stopping (compatible window)	+3
Drug present in body on index day	Short half‐life (~4 h); stopped 5 days prior, by more than 5 times the elimination half‐life	−1
Pre‐challenge/Re‐challenge	None	0
De‐challenge	Improvement after withdrawal and supportive care	+1
Drug notoriety	Documented reports with fluoroquinolones	+2
Alternative causes	None identified (paracetamol low risk)	0
Total		5 (Probable)

Initial laboratory evaluation showed leukopenia, hyponatremia, and elevated aspartate aminotransferase, while renal function was within normal range. Coagulation profile was within normal limits, and blood as well as urine cultures were negative (Table 3).

**TABLE 3:** Serial laboratory investigations on admission and follow‐up.

Parameter	April 22	April 23	April 29
CBC and differential			
WBC (× 10^3^/μL)	2.3	2.2	8.8
Neutrophils (%)	62.5	60.4	89.5
Lymphocytes (%)	26.0	23	7.7
Hemoglobin (g/dL)	12.3	12.4	10.5
Hematocrit (%)	37.1	37.4	32.1
MCV (fL)	90.5	90.1	90.9
MCH (pg)	30.0	30.3	29.5
Platelets (× 10^3^/μL)	162	177	376
Renal function			
BUN (mg/dL)	56.3	50	26
Creatinine (mg/dL)	1.00	0.94	0.51
Serum electrolytes			
Sodium (mEq/L)	125	129	—
Potassium (mEq/L)	4.47	4.44	—
Chloride (mEq/L)	105.1	105	—
Liver function			
AST/SGOT (U/L)	122.3	83	97.6
ALT/SGPT (U/L)	32.3	34	49.7
Direct bilirubin	0.126	0.113	0.072
Total bilirubin	0.483	0.354	0.261
Coagulation profile			
PT	12.4	—	—
PTT	25.0	—	—
INR	1.05	—	—
Blood culture	No growth	—	—

The patient was initially resuscitated with a 2 L intravenous bolus of normal saline. Subsequent fluid requirements were calculated according to protocol (2 mL/kg/% body surface area), and a total of 4 L was administered over the next 24 h in the emergency department. The patient was catheterized, with a urine output of 0.8 mL/kg/h. Following initial stabilization, he was transferred to the intensive care unit.

He received omeprazole 80 mg loading dose followed by 40 mg intravenously twice daily. Fentanyl 50 mcg intravenously every 2 h was started for pain control. After multidisciplinary evaluation, treatment was continued with hydrocortisone 50 mg intravenously four times daily, wound care with liquid paraffin, nasogastric tube feeding with a calculated 25 kcal/kg/day, and eye care with saline rinsing and artificial tears twice daily.

## 4 Outcomes and Follow up

On the third hospital day, he developed recurrent hypotension (88/50 mmHg), persistent tachycardia, and a new febrile episode (38.4°C), raising concern for sepsis. Empirical antibiotics consisting of cefepime 1 g intravenously three times daily and vancomycin 1 g intravenously twice daily were initiated and continued for a total of 7 days.

He remained in the intensive care unit for 5 days and subsequently showed clinical improvement. He was transferred to the ward, where the skin lesions evolved into denuded areas with patchy hyperpigmentation and loss of blistering (Figure 1). The patient was discharged in improved condition after 3 weeks.

## 5 Discussion

This case describes a rare instance of near‐total toxic epidermal necrolysis (TEN) induced by ciprofloxacin in a previously healthy 30‐year‐old man. The patient developed characteristic mucocutaneous lesions approximately 5 days after completing a short 5 day course of oral ciprofloxacin 500 mg twice daily prescribed for nonspecific symptoms. The reaction progressed rapidly to involve approximately 80%–90% of the body surface area sparing the scalp and was accompanied by systemic features, including high fever, malaise, and upper gastrointestinal bleeding manifesting as hematemesis. While systemic symptoms are commonly observed in toxic epidermal necrolysis [2, 3] the presence of upper gastrointestinal bleeding in this case represents an unusual and noteworthy feature.

Gastrointestinal bleeding in TEN may result from several mechanisms, including direct mucosal sloughing analogous to skin involvement, stress‐related mucosal disease in critically ill patients, drug‐induced mucosal injury, or coagulopathy associated with systemic inflammation. TEN is also associated with widespread epithelial apoptosis mediated by cytotoxic T cells and inflammatory cytokines, which may also affect the gastrointestinal mucosa, leading to ulceration and bleeding [4, 5]. In this case, the hematemesis may be explained by ongoing mucosal injury and impaired healing due to severe systemic illness.

The presentation was typical of drug‐induced TEN, with prodromal symptoms followed by widespread erythematous macules evolving into blisters, positive Nikolsky sign, extensive epidermal detachment, and involvement of ocular and oral mucosa. What makes this case peculiar is the occurrence of near‐total TEN in a young, otherwise healthy individual with no comorbidities, minimal concomitant medication exposure (only paracetamol, which he had tolerated previously), and development of symptoms after a relatively short course of ciprofloxacin. Most previously reported ciprofloxacin‐associated TEN cases have occurred in patients with underlying conditions such as systemic lupus erythematosus, advanced age, or significant polypharmacy [6, 7]. In our review of the literature, we identified two closely related reports supporting this association. One described life‐threatening gastrointestinal bleeding in the setting of TEN/SJS, including colonic involvement presenting as delayed severe bleeding, suggesting that mucosal injury may evolve even after the initial dermatologic manifestations [8]. Similarly, a more recent case report documented TEN complicated by severe gastrointestinal bleeding in the context of drug‐induced disease, further underscoring the clinical relevance and plausibility of gastrointestinal involvement in TEN [5].

Differential diagnoses considered included staphylococcal scalded skin syndrome, acute generalized exanthematous pustulosis, erythema multiforme major, and other drug‐induced severe cutaneous adverse reactions. However, these were less likely due to the extent of detachment (> 30% BSA), prominent mucosal involvement, and absence of typical features such as Nikolsky‐negative findings or pustular eruptions. Infectious causes were excluded by negative cultures and clinical course. Paracetamol was deemed very unlikely based on prior uneventful exposure and incompatible timing. The strong temporal relationship (onset within the classic 4–28 day latency window), characteristic clinical features, and ALDEN score of 5 (probable) made ciprofloxacin the most likely culprit [9].

Management in this case followed established international guidelines emphasizing immediate withdrawal of the suspected drug, aggressive supportive care in the intensive care unit, fluid and electrolyte management, nutritional support, meticulous wound care, and prevention of secondary infection [1, 2]. Short‐course systemic corticosteroids (hydrocortisone) and proton pump inhibitors for gastrointestinal protection were administered. Empirical broad‐spectrum antibiotics were added appropriately on Day 3 when sepsis was suspected. Despite the extensive skin involvement, the patient showed progressive improvement and survived, contrasting with several fatal ciprofloxacin‐induced TEN cases reported in the literature [10].

This case adds to the growing but still limited body of evidence linking fluoroquinolones to severe cutaneous adverse reactions. While sulfonamides, allopurinol, and aromatic anticonvulsants remain the highest‐risk drugs, fluoroquinolones such as ciprofloxacin carry a documented lower risk [11]. The successful outcome here underscores the importance of early recognition, prompt drug cessation, and multidisciplinary intensive supportive care, even in resource‐variable settings. This study is limited by the lack of skin biopsy for histopathological confirmation and the unavailability of serum drug level monitoring. Genetic testing was not performed due to resource limitations. Despite these constraints, the diagnosis was supported by clinical features and ALDEN causality assessment.

## 6 Conclusion

Ciprofloxacin can rarely trigger life‐threatening near‐total toxic epidermal necrolysis even after short‐term use in otherwise healthy individuals. This case also highlights that upper gastrointestinal bleeding, although uncommon, may occur as an associated manifestation of TEN and can contribute to the clinical presentation. Clinicians should therefore maintain a high index of suspicion not only for severe cutaneous adverse reactions with commonly prescribed antibiotics but also for potential extracutaneous involvement. This report further underscores the utility of the ALDEN algorithm for structured causality assessment and the critical role of prompt drug withdrawal combined with aggressive supportive care in achieving favorable outcomes.

## Author Contributions

**Seid A. abdullahi:** conceptualization, validation, data curation. **Zelalem G. Demissie:** conceptualization, writing – review and editing, validation. **Zeynu H. Endris:** visualization, validation, data curation. **Alemu B. Mesekere:** visualization, validation, data curation, writing – original draft. **Misale B. Tesfaye:** conceptualization, writing – review and editing. **Tibebu G. Haile:** validation, data curation, visualization. **Amanuel D. Wakoya:** writing – review and editing, writing – original draft, conceptualization.

## Funding

The authors have nothing to report.

## Ethics Statement

The authors have nothing to report.

## Consent

Written informed consent was obtained from the patient for publication of this case report and use of images. A copy of the written consent is available for review by the Editor‐in‐Chief of this journal upon request.

## Conflicts of Interest

The authors declare no conflicts of interest.

## Data Availability

Data sharing not applicable to this article as no datasets were generated or analysed during the current study.
